# The Effectiveness of Herbal Medicines on Cyclic Mastalgia: A Systematic Review on Meta-analysis

**DOI:** 10.1055/s-0042-1755456

**Published:** 2022-11-29

**Authors:** Firoozeh Mirzaee, Farzaneh Rashidi Fakari, Masoudeh Babakhanian, Nasibeh Roozbeh, Masumeh Ghazanfarpour

**Affiliations:** 1 Department of Midwifery, Nursing Research Center, Kerman University of Medical Sciences, Kerman, Iran; 2 Department of Midwifery, School of Medicine, North Khorasan University of Medical Sciences, Bojnurd, Iran; 3 Social Determinants of Health Research Center, Semnan University of Medical Sciences, Semnan, Iran; 4 Department of Midwifery, University of Medical Sciences, Bandar Abbas, Iran

**Keywords:** mastodynia, phytoestrogens, systematic review, herbal medicine, mastodinia, fitoestrogênios, revisão sistemática, fitoterapia

## Abstract

**Objective** Different drugs are used to treat mastalgia, such as danazol and bromocriptine, and both are associated with side effects, due to which most of women and healthcare providers are interested in herbal medicines. Therefore we aim to study the effectiveness of phytoestrogens on the severity of cyclic mastalgia.

**Methods** To carry out the present study, English electronic resources such as the Cochrane Library, ISI Web of Science, Scopus, and PubMed were used systematically and with no time limitation up to February 10, 2020.

**Results** In total, 20 studies were included in the present meta-analysis. The results of the meta-analysis showed that herbal medicines versus the control group (standard mean difference [SMD] = - 0.585; 95% confidence interval [CI]: - 0.728–- 0.44; heterogeneity; *p* = 0.02; I2 = 42%), herbal medicines versus the B group (SMD = - 0.59; 95%CI: - 0.75–- 0.44; heterogeneity; *p* = 0.03; I2 = 42%), and its subgroups, such as phytoestrogen (SMD = - 0.691; 95%CI: - 0.82–- 0.55; heterogeneity; *p* = 0.669; I2 = 0%), Vitex-agnus-castus (SMD = - 0.642; 95%CI: - 0.84–- 0.44; *p* < 0.001; *p* = 203; I2 = 32%), flaxseed (SMD = - 0.63; 95%CI: - 0.901–- 0.367; *p* = 0.871; I2 = 0%), and evening primrose (SMD= - 0.485; 95%CI:- 0.84–- 0.12; *p* = 0.008; heterogeneity; *p* = 0.06; I2 = 56%] may have effective and helpful effects on improving cyclic breast mastalgia. Also, chamomile, isoflavone, cinnamon, and nigella sativa significantly reduced mastalgia symptoms.

**Conclusion** Herbal medicines and their subgroups may have effective and helpful effects on improving cyclic breast mastalgia. The findings of our meta-analysis must be done cautiously because low methodological quality in some evaluated studies of this systematic review.

## Resumo

**Objetivo** Diferentes drogas são utilizadas para tratar a mastalgia, como danazol e bromocriptina, e ambas estão associadas a efeitos colaterais, devido aos quais a maioria das mulheres e dos profissionais de saúde está interessada em medicamentos fitoterápicos. Portanto, nosso objetivo no presente estudo é estudar a eficácia dos fitoestrogênios na gravidade da mastalgia cíclica.

**Métodos** Para a realização do presente estudo, foram utilizados recursos eletrônicos em inglês como a Cochrane Library, ISI Web of Science, Scopus e PubMed, de forma sistemática e sem limitação de tempo até 10 de fevereiro de 2020.

**Resultados** No total, 20 estudos foram incluídos na presente metanálise. Os resultados da metanálise mostraram que fitoterápicos *versus* grupo controle (SMD = - 0,585; intervalo de confiança (IC) 95%: - 0,728–- 0,44; heterogeneidade; *p* = 0,02; I2 = 42%), fitoterápicos *versus* grupo B (SMD = - 0,59; IC95%: - 0,75–- 0,44; heterogeneidade; *p* = 0,03; I2 = 42%) e seus subgrupos, como fitoestrogênios (SMD = - 0,691; IC95%: - 0,82–- 0,55; heterogeneidade; *p* = 0,669; I2 = 0%), Vitex-agnus-castus (SMD = - 0,642; IC95%: - 0,84–- 0,44; *p* < 0,001; *p* = 203; I2 = 32%), linhaça (SMD = - 0,63; IC95%: - 0,901–- 0,367; *p* = 0,871; I2 = 0%) e prímula (SMD = - 0,485; IC95%: - 0,84–- 0,12; *p* = 0,008; heterogeneidade; *p* = 0,06; I2 = 56%) podem ter efeitos eficazes e úteis na melhora da mastalgia cíclica da mama. Além disso, camomila, isoflavona, canela e Nigella sativa reduziram significativamente a mastalgia.

**Conclusão** Os medicamentos fitoterápicos e seus subgrupos podem ter efeitos eficazes e úteis na melhora da mastalgia mamária cíclica. Os achados do presente estudo devem ser explantados com atenção devido ao pequeno número de estudos existentes sobre o tema, a maioria dos quais com um tamanho de amostra pequeno.

## Introduction

Breast pain may be divided in two major categories: cyclic pain and noncyclic pain. 1 2 Cyclic breast pain exacerbates with the onset of the second half of the menstrual period and alleviates with the onset of menstrual bleeding; it is also distributed bilaterally toward the upper arms and armpits. 3 It may last > 5 days and ∼ 30% of women with mastalgia 4 and 11% of women may suffer from pain for 7 days. There are different studies on the prevalence of cyclic breast pain, which has been reported to range from 30 to 70%. 3 Breast pain may provoke anxiety and concern regarding breast cancer among patients; in turn, this concern may impose a high financial burden on the healthcare system due to unnecessary medical referrals and the performance of various diagnostic procedures, such as mammography and biopsy. 5 It also interferes with daily activities, sexual, physical, and social activities. 6 The etiology of breast pain is still unknown. However, the most accepted etiology is related to disturbance in concentration of estrogen, progesterone, and prolactin and the responsiveness of target organs to these hormones Nutritional and psychological causes, water retention in the body, and body and breast weight gain are considered other causes of cyclic mastalgia. 7

Different methods have been used for decreasing mastalgia. Pharmaceutical treatments include danazol, bromocriptine, and tamoxifen, and nonpharmaceutical treatments include supplements, oils, and herbal medicines. 6 Different studies proved that vitamin E is not effective for mastalgia. Although using drugs is associated with excessive expenses, there are also common side effects that renders them inneficient. 8 Meanwhile, tamoxifen has better therapeutic effects and fewer side effects than danazol, so it is mostly used. 9 The use of medicinal plants and herbal medicines has increased recently. Many studies were carried out on the use of herbal medicines for complications of menopause, dysmenorrhea, premenstrual syndrome, mastalgia, etc. 10

Most women, researchers, and healthcare providers have been interested in herbal medicines and phytoestrogens. Phytoestrogens are some compounds that are similar to 17-β-Sterol in terms of structure and function, or may have some effects similar to estrogens. 11 Phytoestrogens include several groups of compounds such as lignans, isoflavones, and coumestans. 12 There is much research on the effects of phytoestrogens on the severity of cyclic mastalgia. 13 14 15 16 Currently, danazol is used as the only effective treatment licensed for mastalgia associated with side effects. Tamoxifen as a third-line therapy is not currently licensed for breast pain treatment. 17 We have identified new studies that met the inclusion criteria that were not included in the previous systematic reviews. The purpose of the present study was to investigate the effectiveness of phytoestrogens on the severity of cyclic mastalgia.

## Methods

English electronic resources such as ISI Web of Science, Scopus, PubMed, and Cochrane Library were used systematically and with no limitations up to February 10, 2020, in order to carry out the present study. The following keywords were used to find out research articles related to the effects of herbal medicines on cyclic mastalgia: ( *Mastalgia* ) and ( *Complementary treatments* OR *alternative treatments* OR *phytomedicine* OR *herbal treatments* OR *alternative medicine* OR *complementary medicine* OR *Vitex agnus-castus* OR *chaste* OR *flaxseed* OR *isoflavones* OR *soy* OR *Matricaria chamomilla* OR *chamomile* OR *Nigella Sativa* OR *Cinnamon* . The references of the included articles and review articles on the subject of the present study were also carefully reviewed to complete the search. The search results from these five databases were merged and duplicates were deleted (based on the same title, year of publication, and name of the author).

Two authors independently investigated the title and abstract of articles, and the complete articles were extracted and investigated when they found that the subject is related to the purpose of the current research.

All clinical trials investigated the effect of oral or topical herbal therapies in the treatment of cyclic or noncyclic mastalgia. The intervention included women receiving herbal medicines as monotherapy or in combination with other chemical or herbal medications. Placebo, herbal medicine, chemical medication, usual care, and no interven tion considered as control group.

We also excluded conference papers, review papers, Editor's Notes, letters, case reports, and animal studies. In cases in which several reports from a study appeared to have been published, only one with more complete information was included, and the others were deleted. These cases were identified by controlling the similarity of the team of authors, the center and the period of the study, and the reported statistical results.

The selection of related articles was carried out by two independent reviewers within two steps. In the screening phase, the titles were read first, and a decision was made to enter the analysis. In case of any ambiguity in the inclusion of the article, the abstracts were reviewed to match their title and abstract with the inclusion and exclusion criteria. Cases that were suspicious and required to be fully read entered the second stage. In the second phase, the full text of the reviewed articles and the articles that fully complied with the inclusion and exclusion criteria were entered into a systematic review. All included articles, review articles, and references of articles on the study subject were also carefully reviewed to complete the search.

The data extraction table was designed by the research team and each article in the present study was reviewed by two independent researchers. The following data were extracted and reported in the table: Authors, country, age of the patients, duration of treatment, number of subjects submitted to the intervention, type of control of the intervention, and assessment tool results ( Chart 1 ).

**Chart 1:** Specifications of the studies included in the present systematic review article

Author (year)	Type of clinical trial	Age (years old)	Outcome	Intervention (dose and duration of treatment)	Comparison (dose and duration of treatment)	Duration of follow-up	Intensity of mastalgia	Assessment tools	Inclusion criteria	Results
Vaziri et al. (2014) 16	Single-blind	20–45	Treatment of mastalgia	180 g of flaxseed for 2 cycles	Omega 3 fatty acids (180 mg of eicosapentaenoic acid and 120 mg of docosahexaenoic acid)	3 months	Cyclicmastalgia	Visual analog scale	181	Flaxseed was more effective in reducing mastalgia
Sekhavat et al. (2009) 18	Double-blind	18–40	Treatment of mastalgia	60 drops of Vitagnus daily	Placebo	3 months	Cyclic or noncyclic mastalgia	Visual analog scale	117	Vitagnus reduced mastalgia more than placebo.
Saghafi et al. (2018) 15	Double-blind	> 18	Treatment of mastalgia	5 drops of chamomile 3 times a day for 2 consecutive months	Placebo(distilled water)	2 months	Cyclicmastalgia	Visual analog scale	55	Chamomile reduced mild to moderate mastalgia.
Rajaby Gharaiy et al. (2017) 13	Double-blind	18–40	Treatment of mastalgia	400 mg of cinnamon 3 times a day	Placebo	2 months	Cyclic mastalgia	Cardiff checklist	74	Cinnamon can be effective in reducing the severity of mastalgia in women.
Mirmolaei et al. (2017) 14	Triple-blind	15–49	Treatment of mastalgia	10 ml (2 tablespoons) of Nigella sativa syrup	Placebo(paraffin oil syrup)	2 months	Cyclic mastalgia	McGill questionnaire and visual analog scale	72	Nigella sativa syrup reduced pain intensity compared with placebo.
Jahdi et al. (2019) 19	Triple-blind	18–50	Treatment of mastalgia	1000 mg evening primrose every 12 hours, 50 mg vitamin B6 every 12 hours,	Placebo	1, 2, and 3 months	Cyclic mastalgia	Visual analog scale	94	B6 and evening primrose have the same therapeutic effects in the treatment of cyclical mastalgia
Alvandipour et al. (2011) 9	Double-blind	–	Treatment of mastalgia	Evening primrose 2 g/day and vitamin E 400 mg/day	Placebo	After 1 month and 6 months	Cyclic mastalgia	McGill questionnaire	100 women with cyclic mastalgia	Evening primrose and vitamin E had a similar effect in the treatment of mastalgia
Gateley et al. (1992) 20	Clinical trial	> 17	Treatment of mastalgia	Danazol 200 mg daily/bromocriptine 1.25 mg daily	Evening primrose oil, 3 g/day	2 months	Cyclicmastalgia	Cardiff checklist	478 women with cyclic mastalgia	Danazol was more effective in reducing the severity of mastalgia in women than bromocriptine and evening primrose oil.
Blommers et al. (2002) 21	Double-blind clinical trial	−	Treatment of mastalgia	3 g of evening primrose oil and control oil	3 g of fish oil and control oil	3 and 6 months	Cyclic or noncyclic mastalgia	Clinical examinations	120 women with cyclicmastalgia	Both groups showed a similar reduction in pain.
Aydin et al. (2012) 22	Prospective clinical trial	19–54	Treatment of mastalgia	Group 1 = vitex agnus castus and group 2 = meloxicam	Placebo	3months	Cyclic mastalgia	Visual analog scale	108 women with cyclicmastalgia for at least 5 days in 1 cycle with normal and high prolactin	Vitex-agnus-castus was more effective in reducing mastalgia than meloxicam and placebo.
Jaafarnejad et al. (2017) 23	Quasiexperimental clinical trial	18–45	Treatment of mastalgia	Group 1 = flaxseed, group 2 = 1000-mg capsules of evening primrose oil daily	Vitamin E group, 400-IU capsules	1–2 months	Cyclic mastalgia	Researcher-made checklist	Women with cyclicmastalgia	Flaxseed, evening primrose oil, and vitamin E reduced the duration of mastalgia, but this decrease was significant only inin the flaxseed group.
Ingram et al. (2002) 24	Double-blind	>18	Treatment of mastalgia	Isoflavones, 80 and 40 mg daily	Placebo	2 months	Cyclic mastalgia	Breast pain checklist	12 women with cyclicmastalgia	Isoflavones could be effective as complementary therapy in the treatment of mastalgia.
Mirghafourvand et al. (2016) 2	Double-blind	18–45	Treatment of mastalgia	Group 1 = 25 g flaxseed powder and group 2 = 3.2–8.8 mg of Vitagnus daily	Placebo	2 months	Cyclic mastalgia	Cardiff checklist	159 women with cyclical mastalgia	Flaxseed and Vitagnus were effective in reducing mastalgia in the short term.
Kiliç et al. (2016) 25	Prospective clinical study	> 18	Treatment of mastalgia	Group 1 = evening primrose oil and group 2= fructus agni casti/reassurance	Placebo	3 months	Cyclic or noncyclic mastalgia	Cardiff checklist	128 women with cyclicmastalgia	Fructus agni casti was more effective in reducing mastalgia than evening primrose and placebo.
Ataollahi et al. (2015) 26	Triple-blind	–	Treatment of the symptoms of premenstrual syndrome	400 g wheat germ 3 times a day from the 16th day of the cycle until the next 5 periods	Placebo	2 months	Cyclic mastalgia	Daily Symptom Record	84 women with premenstrual syndrome	Wheat germ was effective in treating mastalgia
Ghazanfarpour et al. (2011) 27	Double-blind	31	Treatment of the symptoms of premenstrual syndrome	Hypericum perforatum (1360-μg hypericin tablets per day)	Placebo	2 months	Undermine	Premenstrual syndrome questionnaire	170	Hypericum perforatum was more effectiveness than placebo
Pruthi et al. (2010) 28	Double-blind	> 18	Treatment of mastalgia	3 g of evening primrose	Placebo	6 months	Mastalgia	McGill questionnaire	85 women > 18 years old who develop mastalgia for at least 2 cycles 2 weeks before menstruation	Evening primrose and vitamin E, either alone or in combination, had a similar effect in the treatment of mastalgia
Masumi et al. (2017) 29	Double-blind	> 18 years	–	1000 mg of evening primrose daily	400 mg of vitamin E daily	60 days	Undermine	Premenstrual Symptoms Screening Tool	70 women with moderate to severe menstrual syndrome	Evening primrose caused a greater decrease in the treatment of premenstrual syndrome symptoms than vitamin E.
Pakgohar et al. (2005) 30	Double-blind	–	Treatment of premenstrual syndrome	60 drops of Hypiran daily 7 days before menstruation for 2 cycles	Placebo (60 drops daily 7 days before menstruation in 2 cycles)	2 months	Undermine	Daily Symptom Record	70 students with at least 5 symptoms of premenstrual syndrome	Hypiran was more effective than placebo in treating the symptoms of premenstrual syndrome, including mastalgia.
Mirmolaei et al. (2017) 14	Triple-blind	15–49	Treatment of mastalgia	Daily Vitagnus (8 cc)	Placebo (Oral paraffin)	3 months	Cyclic mastalgia	McGill questionnaire and visual analog scale	67 women aged 15 to 49 years old with a visual analog scale score > 4	Vitagnus was more effective in reducing mastalgia than placebo.

## Evaluating the Quality of Articles

The Final Jadad scale including three items was used to evaluate the quality of articles. 31 These items was considered in terms of randomization (whether randomization was done and whether it was done appropriately), blinding (whether the trial was blinded and whether it was done appropriately), reporting account of all patients ( Chart 2 ).

**Chart 2:** Assessment of the quality of studies by the Jadad Scale

Authors	Blinding			Randomization			Account of all patients
	Mentions randomization	Method: appropriate	Method: inappropriate	Mention srandomization	Method: appropriate	Method: inappropriate	
Vaziri et al. (2014) 16	+	+	−	−	−	−	+
Sekhavat et al. (2009) 18	+	+	−	+	+	−	+
Saghafi et al. (2018) 15	+	+	−	+	+	−	+
Rajaby Gharaiy et al. (2017) 13	+	+	−	+	+	−	+
Mirmolaei et al. (2017) 14	+	+	−	+	+	−	+
Jahdi et al. (2019) 19	+	+	−	+	+	−	+
Alvandipour et al. (2011) 9	+	+	−	−	−	−	+
Gateley et al. (1992) 20	+	+	−	+	+	−	−
Aydin et al. (2012) 22	−	−	−	−	−	−	−
Blommers et al. (2002) 21	+	+	−	+	−	+	+
Jaafarnejad et al. (2017) 23	+	−	−	−	−	−	+
Ingram et al. (2002) 24	−	+	+	+	+	−	+
Mirghafourvand et al. (2016) 2	+	+	−	+	+	−	+
Kiliç et al. (2016) 25	+	−	−	−	−	−	−
Ataollahi et al. (2015) 26	+	+	−	+	−	−	−
Ghazanfarpour et al. (2011) 27	+	+	−	+	+	−	+
Pruthi et al. (2010) 28	+	+	−	+	+	−	+
Masumi et al. (2017) 29	+	+	−	+	+	−	+
Pakgohar et al. (2005) 30	+	+	−	+	+	−	+
Mirmolaei et al. (2016) 10	+	+	−	+	+	−	+

### Statistical Analysis

The software Comprehensive Meta-analysis (CMA) version 2 (Biostat Inc. Englewood, NJ, USA) was used to perform the data analysis. The heterogeneity index of studies was determined by the I2 test and the Q Cochran test. According to the results of Higgins et al. 32 , it is considered that values < 25% show low heterogeneity; values between 25 and 75% show moderate heterogeneity; and values > 75% shows high levels of heterogeneity. According to the results of heterogeneity, random was used to report the effect of phytoestrogens if heterogeneity was 25 percent or higher instead of fixed effect. Forest plot was used to demonstrate the results of the meta-analysis in which the size of the squares shows the number of samples of each, and lines drawn on both sides show the 95% confidence interval (CI) for the effects of each study.

## Results

### Herbal Medicines versus Control Group

**Fig. 1:**
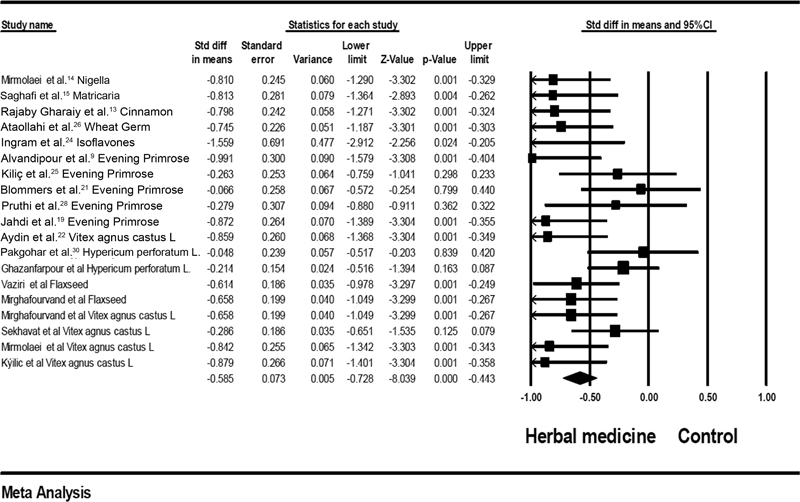
Effects of herbal medicines versus control on mastalgia. The horizontal lines denote the 95% confidence interval; □ point estimate (size of the square corresponds to its weight); ♦, combined overall effect of treatment

The results of the Q Cochran test demonstrate the heterogeneity between the results of the different studies and a random model of meta-analysis was used instead of a fixed model ( *p* = 0.02; I2 = 42%). The standardized mean difference (SMD) value between the intervention group and the control group was SMD = - 0.585; 95%CI: - 0.728–- 0.44; heterogeneity; *p* = 0.02; I2 = 42%) ( Fig. 1 ), with statistical significance ( *p* < 0.001). The findings showed that the severity of the pain was lower in the herbal medicine group in comparison with the control group ( *p* < 0.001). 2 9 10 13 14 15 16 18 19 21 22 24 25 26 28 30

### Herbal Medicines versus Placebo

**Fig. 2:**
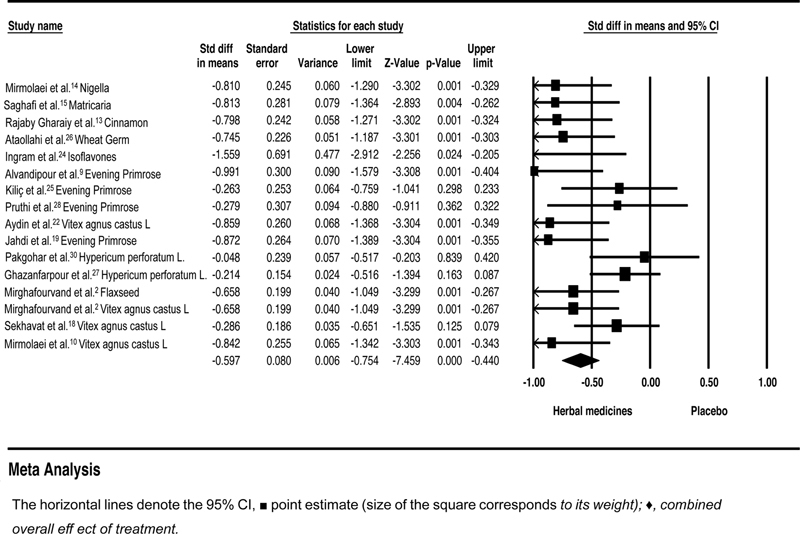
Effects of herbal medicines versus placebo on mastalgia. The horizontal lines denote the 95% confidence interval; □ point estimate (size of the square corresponds to its weight); ♦, combined overall effect of treatment

The SMD value between the herbal medicines group and the placebo group was SMD = - 0.59; 95%CI: - 0.75–- 0.44; heterogeneity; *p* = 0.03; I2 = 42% ( Fig. 2 ). The heterogeneity between the studies was moderate. Sensitivity analysis was conducted based on the type and severity of mastalgia. 2 9 10 13 14 15 18 19 22 24 25 26 28 30

**Fig. 3:**
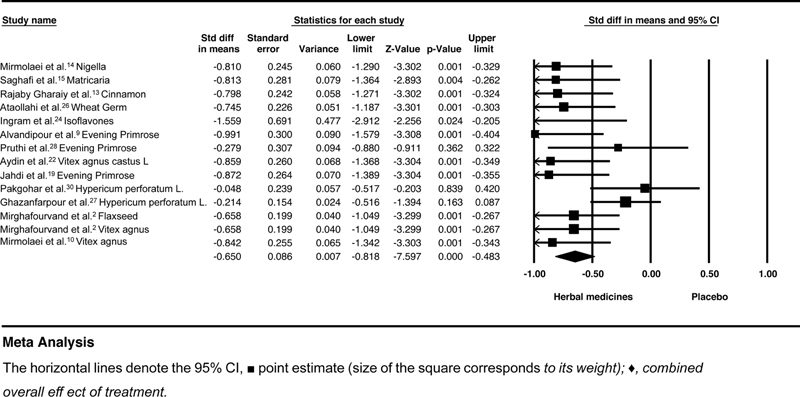
Effects of herbal medicines versus placebo on cyclical mastalgia. The horizontal lines denote the 95% confidence interval; □ point estimate (size of the square corresponds to its weight); ♦, combined overall effect of treatment.

The intensity of mastalgia was reported mild, therefore Sensitivity analysis was performed to exclude Saghafi et al. 15 The SMD and heterogeneity did not change after the removal of Saghafi study (SMD = - 0.58; 95%CI: - 0.75–- 0.42; heterogeneity; *p* = 0.03; I2 = 44%; random effect model). The second sensitivity analysis was performed to exclude studies that reported both cyclical and noncyclical mastalgia. The SMD values increased from - 0.59 to 0.65, and heterogeneity was slightly reduced to 40% (SMD = - 0.65; 95%CI: - 0.81–- 0.48; heterogeneity; *p* < 0.001; I2 = 40%; *p* = 0.059; random effect model) ( Fig. 3 ). 2 9 10 13 14 15 19 22 24 26 28 30

### Phytoestrogen versus Control

**Fig. 4:**
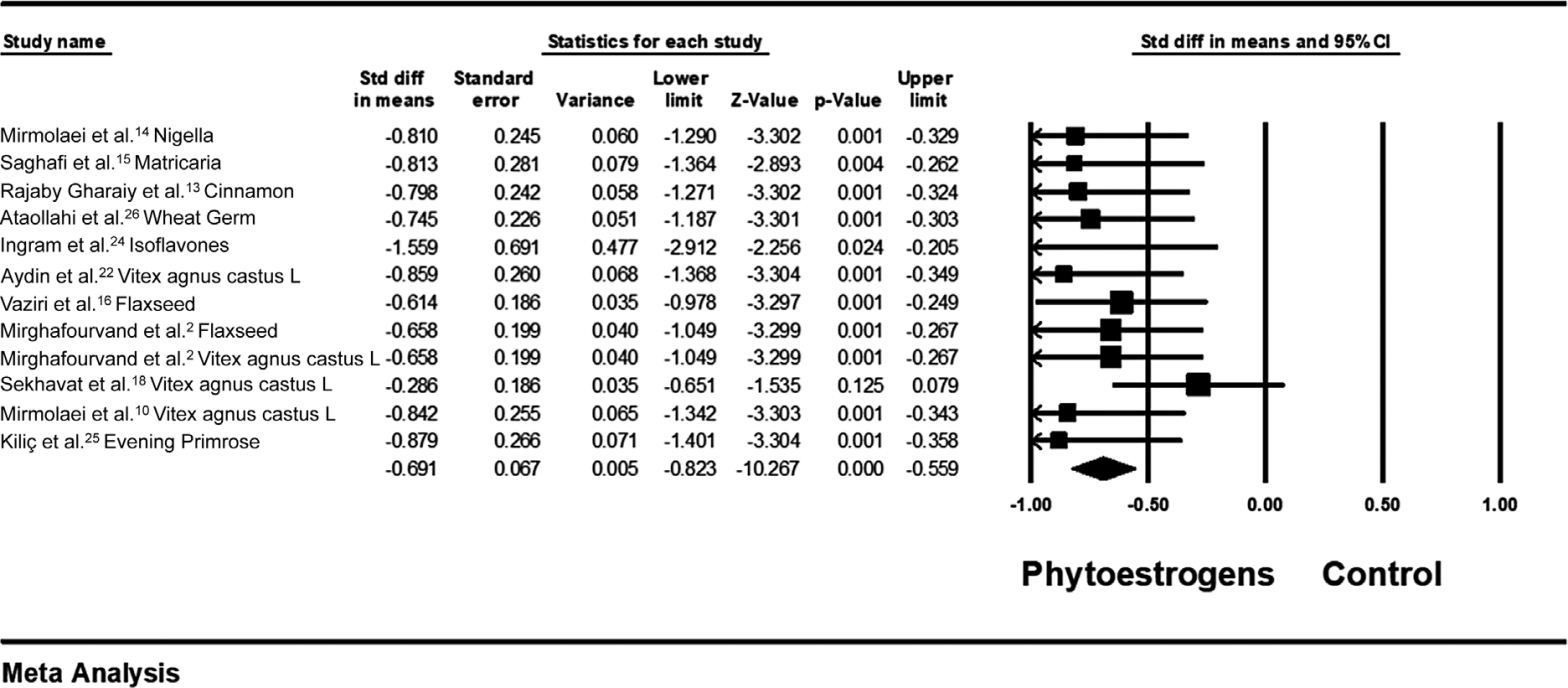
Effects of phytoestrogens on mastalgia. The horizontal lines denote the 95% confidence interval; □ point estimate (size of the square corresponds to its weight); ♦, combined overall effect of treatment.

The standardized mean difference value between the intervention and control groups was SMD = - 0.691; 95%CI: - 0.82–- 0.55; heterogeneity; *p* = 0.669; I2 = 0%) ( Fig. 4 ). 2 10 13 14 15 16 18 22 24 25 26 27 33 The severity of pain was lower in the phytoestrogen group compared with in the control group ( *p* < 0.001).

### Vitex-agnus-castus versus Control

**Fig. 5:**
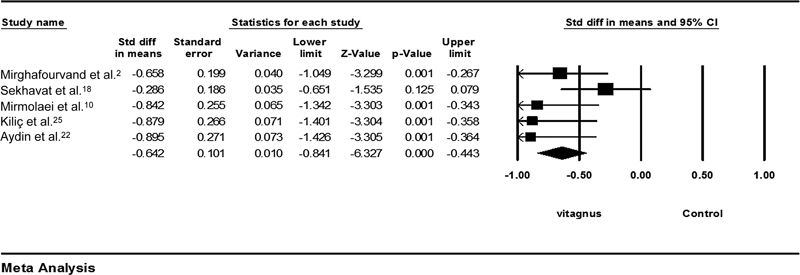
Effects of Vitex-agnus-castus on mastalgia. The horizontal lines denote the 95% confidence interval; □ point estimate (size of the square corresponds to its weight); ♦, combined overall effect of treatment.

The results of the analysis of Vitex-agnus-castus with five studies 2 10 18 22 25 showed that the severity of pain was lower in the Vitex-agnus-castus group compared with in the control group (SMD = - 0.642; 95%CI: - 0.84–-0.44; *p* < 0.001) ( Fig. 5 ). According to the values of the heterogeneity index ( *p* = 203; I2 = 32%), it has been found that there is moderate heterogeneity between studies. Sensitivity analysis was done due to detect potential resource in our meta-analysis. Sekhavat et al. study 18 considered as potential resource heterogeneity and removal of this study decreased heterogeneity to 0%. SMD = 0.793) 95 CI: -1.03 to -0.55; P < 0.001; heterogeneity; p = 0.663; I2 = 0%).

### Flaxseed versus Placebo

**Fig. 6:**
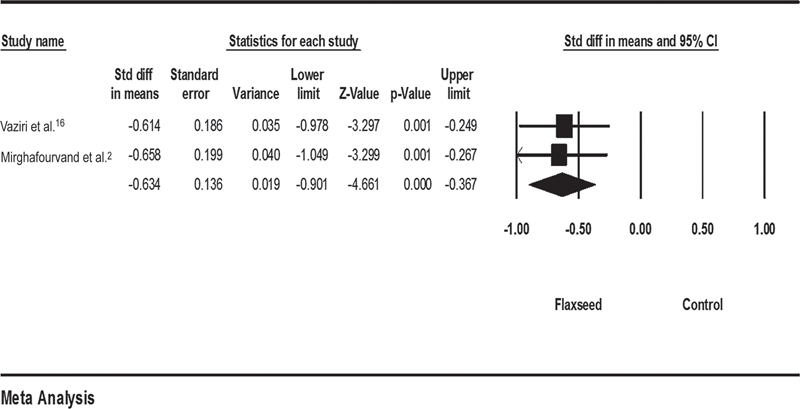
Effects of flaxseed on mastalgia. The horizontal lines denote the 95% confidence interval; □ point estimate (size of the square corresponds to its weight); ♦, combined overall effect of treatment.

The results of analyzing flaxseed with two studies 10 16 showed that women in the flaxseed group reported significantly less pain than those in the control group (SMD = - 0.63; 95%CI: - 0.901–- 0.367; *p* = 0.871; I2 = 0%) ( Fig. 6 ).

### Hypericum Perforatum L. versus Placebo

**Fig. 7:**
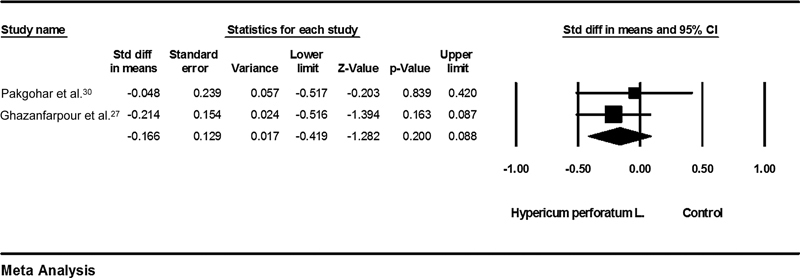
Effects of Hypericum perforatum L. on mastalgia. The horizontal lines denote the 95% confidence interval; □ point estimate (size of the square corresponds to its weight); ♦, combined overall effect of treatment.

The analysis results showed that the effects of Hypericum perforatum L. and placebo were the same in relieving breast pain (SMD = - 0.16; 95%CI: - 0.41–- 0.08; *p* = 0.2; heterogeneity; *p* = 0.55; I2 = 0%; fixed effect model; 2 trials) ( Fig. 7 ). 28 30

### Evening Primrose versus Placebo

**Fig. 8:**
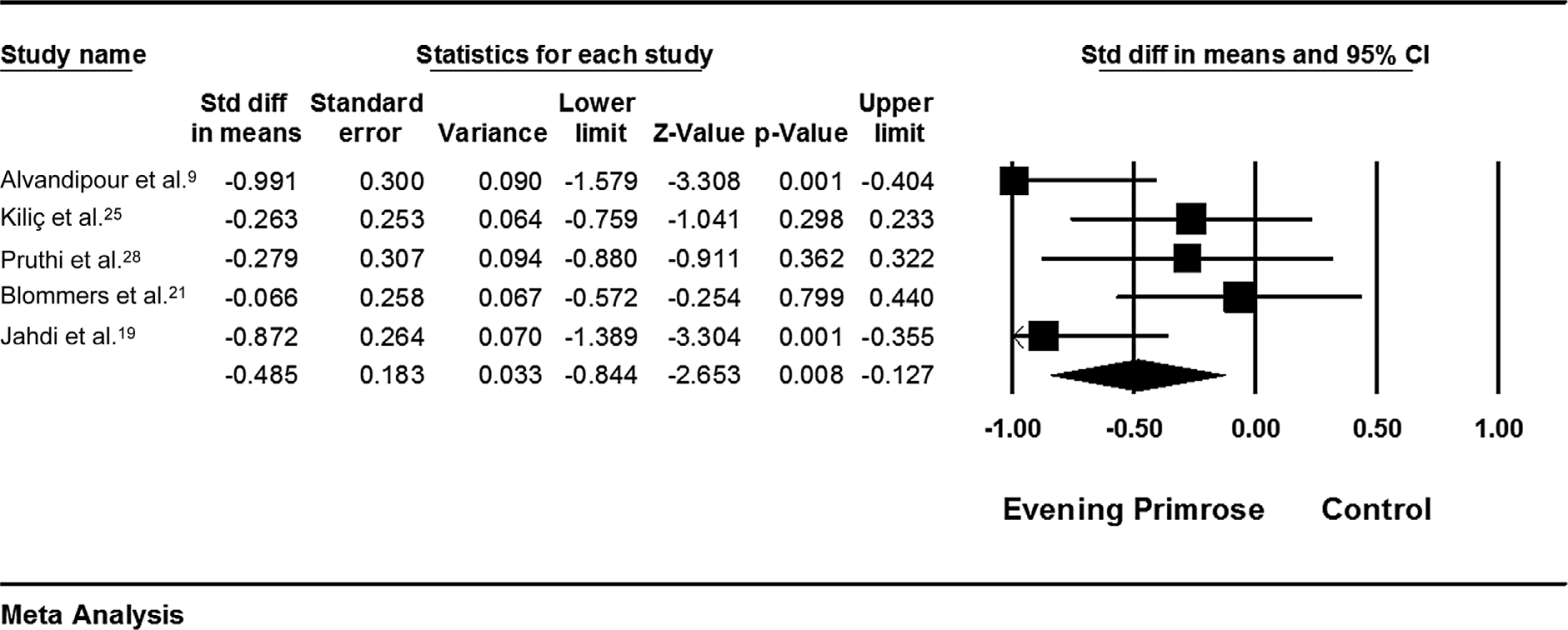
Effects of evening primrose on mastalgia. The horizontal lines denote the 95% confidence interval; □ point estimate (size of the square corresponds to its weight); ♦, combined overall effect of treatment.

The analysis results showed that women in the evening primrose group reported significantly less pain than those in the control group (SMD = - 0.485; 95%CI: - 0.84–- 0.12 ; *p* = 0.008; heterogeneity; *p* = 0.06; I2 = 56%; random effect model) ( Fig. 8 ). 9 19 21 25 28 Sensitivity analysis was conducted due to moderate heterogeneity between studies, and the effect of each study on the final result was evaluated separately. None of the studies had a significant effect on the final result and heterogeneity of the present study.

### Chamomile

A significant reduction in pain was observed in both groups (chamomile and placebo) after 2 months ( *p* < 0.0001 and *p* = 0.048, respectively) compared with baseline and between the two groups ( *p* = 0.007). 15

### Isoflavone

The reduction in pain was 13% for placebo, 44% for 40 mg of isoflavone per day, and 31% for 80 mg per day. There was a significant difference between groups. 24

### Cinnamon

There was a statistically significant difference between the two groups in the mean pain scores at the end of the 1 ^st^ and 2 ^nd^ months ( *p* < 0.001 and *p* = 0.02), meaning that the intensity of the pain at the end of the 1 ^st^ and 2 ^nd^ months were significantly lower in the intervention group than in the control group. 13

### Nigella Sativa

A significant reduction in pain was seen in the Nigella Sativa group compared with the placebo group based on the visual analogue scale (VAS) ( *p* = 0.002). 10

### Evening primrose versus Vitagnus

The mean pain decreased significantly in both the evening primrose ( *p* = 0.004) and in the vitagnus ( *p* < 0.001) groups. Vitagnus was more effective than primrose. The authors did not report a p-value. 33

### Vitamin E versus Flaxseed Oil

Breast pain decreased significantly in both the vitamin E and flaxseed oil groups during the 1 ^st^ and 2 ^nd^ months ( *p* -value among groups < 0.001). However, the mean breast pain was not significantly different between the two groups, which were not different from each other at the end of the 1 ^st^ ( *p* = 0.54) and 2 ^nd^ months ( *p* = 0.73). 34

### Danazol versus Evening Primrose

The overall response with danazol was 76%, in contrast with a 68% response in patients treated with evening primrose. 35 A clinically useful response was observed in 76% cyclical mastalgia and in 36% of those with noncyclical mastalgia treated with danazol, and in 55% of the patients with cyclical mastalgia and in 33% for those with noncyclical mastalgia treated with evening primrose oil. 20

### Evening Primrose versus Bromocriptine

A clinically useful response was observed in 50% of the patients with cyclical mastalgia and in 24% of those with noncyclical mastalgia treated with bromocriptine, and in 55% of the patients with cyclical mastalgia and in 33% of those with noncyclical mastalgia treated with evening primrose oil. 20

### Vitex Agnus Castus with Meloxicam

No significant difference was observed between Vitex-agnus-castus and meloxicam. 22

- The present study showed that GLA (Efamast) efﬁcacy did not differ from that of placebo fatty acids, regardless of whether or not antioxidant vitamins were present.

### Flaxseed and Vitex-agnus-castus

Patients with mastalgia in both the flaxseed and the Vitex-agnus-castus groups reported a significant decrease in breast pain intensity and breast pain length in comparison with placebo ( *p* < 0.01). However, no significant difference was observed between flaxseed and Vitex-agnus-castus in the 1 ^st^ and 2 ^nd^ months. 2

## Discussion

Mastalgia is one of the common problems experienced by women worldwide during reproductive period effects. Drugs like tamoxifen, danazol, and bromocriptine were associated with side effects. As a result, it caused both patients and health providers are interested in herbal medicines. 35 36 According to our investigation, the present research is considered the first meta-analysis on clinical trials that studied the effectiveness of herbal medicines and their subgroups on cyclic mastalgia. Three studies were carried out on vitagnus, 2 10 22 25 one study on nigella sativa, 14 one study on cinnamon, 13 one on isoflavones, 24 two studies on Hypericum perforatum L, 28 30 one study on chamomile, 15 five studies on evening primrose, 9 19 21 25 28 one study on isoflavone, 24 one study compared evening primrose with bromocriptine, 20 vitex agnus castus with meloxicam, 22 and flaxseed with Vitex-agnus-castus. 2 The results of the present research demonstrate that phytoestrogen leads to improvement of cyclic mastalgia compared with placebo. 2 10 13 14 15 18 22 24 25 26 Similarly, nigella sativa, chamomile, cinnamon, and red clover may have helpful effects in improving cyclic mastalgia. According to the result of a study, it can be said that chamomile can significantly reduce the severity of cyclic mastalgia compared with placebo. 15

In vitro, chamomile can inhibit both the function of cyclooxygenase and lipoxygenase; consequently, the production of prostaglandins and leukotrienes is inhibited. 37 This plant is also used as antioxidant, analgesic, antiviral, anti-inflammatory, and antiseptic. 38 According to Gharaiy et al. study, cinnamon is more effective than placebo to reduce the severity of breast pain. 13 Cinnamon contains eugenol, a compound that can prevent prostaglandin biosynthesis and also has anti-inflammatory effects. Research on cinnamon pharmacology and toxicology demonstrate no risk in consuming it. 39

Nigella sativa can relieve breast pain from cyclical mastalgia. 14 This finding is consistent with animal models, as the aqueous extract of N. sativa had anti-inflammatory and analgesic antipyretic effects in albino Wistar rats and albino Swiss mice. 40 Thymoquinone is one of the major compounds of N. sativa, 41 with analgesic, 42 anti-inflammatory, 43 antioxidative, 44 and antioxidative stress effects (Bhandari, 2014). Nigella sativa inhibits inflammatory mediators such as prostaglandins and leukotrienes, macrophage function, NK antitumor activity, amends splenocyte proliferation and Th1/Th2 cytokine profile. 45 The physiological effect of nigella sativa is related to its volatile oil, and thymoquinone is considered one of its active elements with anti-inflammatory effect. 46 The process of inflammation would be regulated by lipoxygenase and cyclooxygenase. Inhibiting lipoxygenase and cyclooxygenase processes prevents the metabolism of arachidonic acid and controls the production of prostaglandins and leukotrienes. 47

Our meta-analysis with studies study shows that flaxseed can reduce the severity of cyclic mastalgia. 2 16 Flaxseed inhibited prostaglandin (PGE2), leukotriene-, histamine-, and bradykinin-induced inflammation, and arachidonic acid-induced inflammation. Flaxseed oil also inhibited both the cyclooxygenase and lipoxygenase pathways of the arachidonate metabolism. 48

Vitex-agnus-castus is known as chaste tree. 49 It is a tree with fingered leaves and cylindrical flowers that grows in the Mediterranean region and Eastern Asia; it has brown fruits which smell of pepper. Its extract is used to treat postpartum hemorrhage. 50 This plant contains progestins, essential oils, diterpenoids, iridoids, flavonoids and ketosteroids. 51 Vitex agnus castus contains iridoids, flavonoids, diterpenoids, and progestins, as well as essential and fatty oils in the fruits, flowers, and leaves. 52 The antiprolactin effect of this plant was evident in previous studies and it is more effective in the treatment premenstrual syndrome. 53 The results of the present meta-analysis showed that, compared with placebo, Vitex-agnus-castus can significantly reduce the severity of cyclic mastalgia. 2 10 18 22 25 The exact mechanism of its effects has not been proved yet but it seems that its effect on the hypothalamus-pituitary axis reduces FSH and prolactin levels and also increases the level of LH. 54 The effect of Vitex Agnus- castus extract on the treatment of luteal phase defects due to latent hyperprolactinemia is investigated. The findings of the study showed a decrease in prolactin levels, normalization of shortened luteal phases duration, and elimination of luteal progesterone. 55 Estimated high to moderate heterogeneity was observed between studies in the evening primrose and Vitex-agnus-castus subgroups, which may be related to different amounts of effective ingredients, different ages of the participants, and different mastalgia severity and pattern of (cyclical and noncyclical). Cyclic mastalgia is more frequent in the 2 ^nd^ and 3 ^rd^ decades of life. 56 Thus, age may have an effect on mastalgia; therefore, to clarify the relationship between age and mastalgia severity, confounders such as age need to be adjusted in future studies. 56

The limited number of studies is a reason for the second limitation of the present study, because it was not possible to evaluate the heterogeneity of studies by metaregression. The third limitation is the level of methodological quality in some evaluated studies of this systematic review was evident. The problems we have observed in these studies were inappropriate methods of randomization and double-blinding and lack of reportingintention-to-treat analysis. It is suggested that future studies future studies should adhere to CONSORT guidelines. The limited number of studies and their small sample sizes are the fourth imitation of the present study, and more studies with larger sample sizes are warranted. The fifth limitation is that some of the studies have a small sample size, and any enhancement in sample size may have changed the results of these studies. Some studies had no placebo group, so they were not included in the present study. Some studies were designed with test and post-test with no control group and were excluded from the present study, so we suggested designing future studies with placebo and control groups. The sixth limitation is that phytoestrogens are divided into four groups (isoflavones, gensitin, dydizin, and glycine). We suggest that future studies investigate the effect on cyclic mastalgia of other phytoestrogen compounds in the aforementioned groups so that their results can help us to reach a better understanding of their function. The seventh limitation is that generalizing the research findings must be done cautiously because all of the studies were conducted in Iran. The length of treatment was short in most studies and therapeutic effects have not been evaluated after discontinuing the drugs. Finally, all phytoestrogens evaluated in the present meta-analysis had positive effects on the severity of mastalgia, but the function of these phytoestrogens was not investigated in any of the included studies. We recommend the investigation of this important issue in future studies. Since phytoestrogens have a positive effect on cyclic mastalgia, we suggest investigating the effect of phytoestrogens on noncyclic mastalgia; further studies with longer duration and with a follow-up phase should be performed in order to investigate the maintenance of their effectiveness. Some studies have reported that such an assurance the women obtain when clinical breast examination can effect on the intensity of cyclical mastalgia 16 the confounding variables should be controlled in future study.

## Conclusion

Due to the important effect of the health of women in their function in the family and in society and to the fact that mastalgia may cause disruption on their activities and also the positive effect of the effectiveness of herbal medicines, this study was peformed to investigate the effectiveness of herbal medicines on the severity of cyclic mastalgia. The findings of this study showed that herbal medicines such as nigella sativa, chamomile, flaxseed, vitex-agnus-castus and red clover can be considered as an effective and helpful method in improving cyclic mastalgia. The findings of the included studies must be interpreted cautiously due to the high level of heterogeneity between studies, the limited number of studies, and their small sample sizes.
